# Inflammation-induced changes in BK_Ca_ currents in cutaneous dorsal root ganglion neurons from the adult rat

**DOI:** 10.1186/1744-8069-8-37

**Published:** 2012-07-04

**Authors:** Xiu-Lin Zhang, Lee-Peng Mok, Kwan Yeop Lee, Marcel Charbonnet, Michael S Gold

**Affiliations:** 1 Department of Anesthesiology, University of Pittsburgh, 3500 Terrace Street Rm E1440 BST, Pittsburgh, PA, 15213, USA; 2 Department of Neurobiology, University of Pittsburgh, Pittsburgh, PA, USA; 3 Department of Medicine, Division of Gastroenterology Hepatology and Nutrition, Pittsburgh, PA, USA; 4 Pittsburgh Center for Pain Research, University of Pittsburgh, Pittsburgh, PA, USA; 5 Department of Biomedical Sciences, University of Maryland, Baltimore, MD, USA

**Keywords:** Sensitization, Voltage clamp, Nociceptor, Perforated patch, *in vitro*

## Abstract

**Background:**

Inflammation-induced sensitization of primary afferents is associated with a decrease in K^+^ current. However, the type of K^+^ current and basis for the decrease varies as a function of target of innervation. Because glabrous skin of the rat hindpaw is used often to assess changes in nociception in models of persistent pain, the purpose of the present study was to determine the type and extent to which K^+^ currents contribute to the inflammation-induced sensitization of cutaneous afferents. Acutely dissociated retrogradely labeled cutaneous dorsal root ganglion neurons from naïve and inflamed (3 days post complete Freund’s adjuvant injection) rats were studied with whole cell and perforated patch techniques.

**Results:**

Inflammation-induced sensitization of small diameter cutaneous neurons was associated with an increase in action potential duration and rate of decay of the afterhyperpolarization. However, no changes in voltage-gated K^+^ currents were detected. In contrast, Ca^2+^ modulated iberiotoxin sensitive and paxilline sensitive K^+^ (BK_Ca_) currents were significantly smaller in small diameter IB4+ neurons. This decrease in current was not associated with a detectable change in total protein levels of the BK_Ca_ channel α or β subunits. Single cell PCR analysis revealed a significant change in the pattern of expression of α subunit splice variants and β subunits that were consistent, at least in part, with inflammation-induced changes in the biophysical properties of BK_Ca_ currents in cutaneous neurons.

**Conclusions:**

Results of this study provide additional support for the conclusion that it may be possible, if not necessary to selectively treat pain arising from specific body regions. Because a decrease in BK_Ca_ current appears to contribute to the inflammation-induced sensitization of cutaneous afferents, BK_Ca_ channel openers may be effective for the treatment of inflammatory pain.

## Background

Peripheral inflammation is associated with pain and hyperalgesia that reflects, at least in part, the sensitization of primary afferents innervating the site of inflammation [1]. This increase in excitability reflects both acute (i.e., phosphorylation) and persistent (i.e., transcription) changes in a variety of ion channels [1] that control afferent excitability. Results from a series of studies on afferents innervating glabrous skin of the rat suggest that the impact of inflammation on the underlying mechanisms of sensitization is complex. Analysis of afferents *in vivo* indicate that the inflammation-induced increase in excitability is associated with changes in axon conduction velocity, [2] as well as changes in the action potential waveform invading the cell soma in a subpopulation of afferents [3]. Evidence from a relatively small subpopulation of acutely dissociated cutaneous sensory neurons *in vitro*, suggest that at least some of the changes observed *in vivo* are due to changes intrinsic to the sensitized afferents [4]. This observation is consistent with the suggestion that changes in the density, distribution and/or expression of ion channels contributes to the inflammation-induced increase in excitability. Persistent inflammation is also associated with at least two changes in Ca^2+^ signaling in cutaneous neurons which include an increase in the magnitude and duration of depolarization-induced Ca^2+^ transients [5] and a decrease in the density of high threshold voltage-gated Ca^2+^ current [6]. In the latter study, there is a subpopulation of neurons in which a decrease in high threshold Ca^2+^ current resulted in an increase in excitability, presumably secondary to a decrease in Ca^2+^-modulated K^+^ current, and a second population in which a decrease in Ca^2+^ current was associated with a decrease in excitability. These results were consistent with the observation that Ca^2+^ modulated iberiotoxin (IbTx) sensitive and paxilline sensitive K^+^ (BK_Ca_) currents are differently distributed among subpopulations of cutaneous afferents [7,8]. Furthermore, because multiple splice variants of the α subunit and 3 of the 4 β subunits of the BK_Ca_ channel underlying this current was detected in mRNA extracted from L4 and L5 dorsal root ganglia (DRG) [8], the impact of inflammation-induced changes in Ca^2+^ signaling on afferent excitability will therefore depend on BK_Ca_ channel splice variants and β subunits as well as the proximity of these channels to the sources of intracellular Ca^2+^. Finally, we and others have demonstrated that persistent inflammation of other tissues including the masseter muscle [9], colon [10], bladder [11] and stomach [12] is associated with a decrease in voltage-gated K^+^ current. Thus, there is the possibility that a decrease in at least two K^+^ currents contributes to persistent inflammation-induced sensitization of cutaneous afferents.

Nevertheless, because of evidence that the specific K^+^ current changes associated with persistent inflammation depend on the target of innervation [9] and because of the changes in Ca^2+^ signaling in cutaneous afferents associated with persistent inflammation, we hypothesize that changes in a Ca^2+^-dependent K^+^ current is primarily responsible for the sensitization of cutaneous afferents. To test this hypothesis, we have analyzed changes in K^+^ currents in cutaneous afferents obtained from naïve and inflamed rats.

## Results

### Sensitization

Based on our previous data indicating the inflammation-induced changes in the regulation of intracellular Ca^2+^[5] and in voltage-gated Ca^2+^ currents [6] is restricted to small and medium diameter cutaneous neurons as well as *in vivo* data suggesting that nociceptive afferents innervating cutaneous tissue tend to have a small cell body diameter [13], we focused on neurons with a cell body diameter <30 μm in the present study. Consistent with results of our previous study, cutaneous neurons from inflamed rats were significantly more excitable than those from naïve rats, where the increase in excitability was manifest with a small but significant decrease in action potential threshold (from −32 ± 0.8 to −34.9 ± 1.0 mV, p = 0.03: n = 53 and 38 for naïve and inflamed groups, respectively), decrease in rheobase (3.7 ± to 2.3 pA/pF, p < 0.05) and increase in the response to suprathreshold current injection (i.e., the number of action potentials evoked in response to current injection 3x rheobase increased from 2.5 ± 0.3 to 6.9 ± 0.9, p < 0.01).

Closer inspection of this dataset, suggested that there were at least two populations of small diameter neurons that could be defined by their response to inflammation: in one there was a clear increase in excitability while in the other, the increase was less apparent. Data suggest that sensory neurons defined by their binding to the lectin IB4 may play a differential role in the hypersensitivity that develops in the presence of inflammation [14,15], We therefore repeated the excitability experiments on neurons incubated in FITC-labeled IB4 10 minutes prior to current clamp recording. The passive electrophysiological properties of the neurons in this dataset were analyzed with a two way ANOVA where IB4 binding and inflammation were the main factors. This analysis revealed a significant (p < 0.01, Table 1) influence of inflammation on membrane capacitance which was increased in cutaneous neurons from inflamed rats. There was also a significant interaction between inflammation and IB4 binding with respect to resting membrane potential where post-hoc analysis indicated that the difference between IB4+ and IB4- neurons in the inflamed group was significant (p = 0.03, Table 1), and the difference between IB4- neurons from the naïve and inflamed groups was also significant (p < 0.05, Table 1).

**Table 1:** Passive Properties of Cutaneous Neurons

Manipulation	IB4	N	V_rest_ (mV)†	R_in_ (MΩ)	Capacitance (pF)‡
Naïve	+	10	-62 ± 2.5	601 ± 199	32 ± 2.8
	-	13	-59 ± 2.2	436 ± 220	31 ± 2.7
CFA	+	9	-59 ± 1.6	800 ± 138	42 ± 1.8
	-	23	-66 ± 2.7	640 ± 220	41 ± 3.0

† Interaction between Manipulation and IB4 binding is significant with p = 0.03.

‡ The impact of Manipulation (inflammation) is significant with p < 0.01.

V_rest_ is resting membrane potential. R_in_ is input resistance. CFA is complete Freund’s adjuvant.

**Figure 1:**
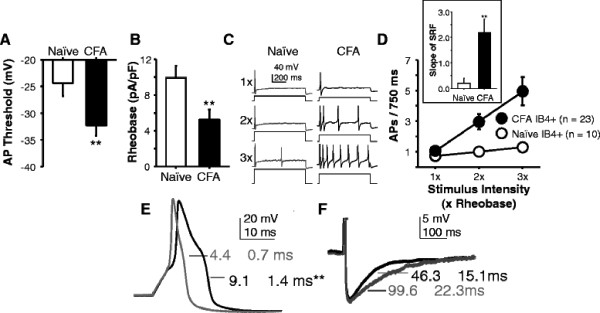
**Persistent inflammation of the hindpaw results in an increase in the excitability of cutaneous neurons that is associated with an increase in action potential duration.** Excitability was assessed in acutely dissociated DRG neurons retrogradely labeled from the glabrous skin of the hindpaw harvested from naïve and inflamed (CFA) rats. Depolarizing current injection was used to determine action potential (AP) threshold (A), rheobase (B) and the response to suprethreshold current injection (C, D), which was injected at intensities 1, 2, and 3 times rheobase. The voltage traces in C, are typical of the pooled data plotted in D. As indicated in D, pooled data for all panels are from 10 neurons from naïve and 23 neurons from inflamed rats. The number of action potentials evoked at 2 and 3x rheobase in neurons from inflamed rats is significantly greater than that in neurons from naïve rats. Error bars for data from naïve neurons are smaller than the symbol. Inset: The slope of the stimulus response function (SRF) is significantly greater for neurons from inflamed rats than that for neurons from naïve rats. E. Typical action potentials from naïve (black trace) and inflamed (gray trace) rats evoked in response to a 4 ms current injection are overlayed with the average action potential duration indicated for each. These values are significantly different (p < 0.05). F. The afterhyperpolarization (AHP) following the action potentials shown in E are plotted to illustrate the trend toward a decrease in the AHP duration in neurons from inflamed rats. The full amplitude of the action potential is clipped in these traces to facilitate visualization of the AHP. Average values are indicated next to each trace, and these differences are significantly different. * is p < 0.05 and ** is p < 0.01

Results of the analysis of the excitability data indicated that there were not only differences between IB4+ and IB4- neurons with respect to baseline excitability, but the changes in excitability 3 days after the induction of inflammation. That is, while the IB4- neurons from naïve rats were more excitable than IB4+ neurons, there were no detectable inflammation-induced changes in the excitability of IB4- neurons: action potential threshold was −35.8 ± 2.3 mV (n = 13) in neurons from naïve rats and −29.0 ± 2.8 mV (p > 0.05, n = 9) in neurons from inflamed rats; rheobase was 4.2 ± 1.4 pA/pF and 6.9 ± 1.6 pA/pF (p > 0.05) in neurons from naïve and inflamed rats, respectively; and the slope of the stimulus response function to suprathreshold current injection was 0.52 ± 0.4 and 0.51 ± 0.5 (p > 0.05) in neurons from naïve and inflamed rats, respectively. In contrast, there was a significant increase in the excitability of IB4+ neurons with a significant decrease in action potential threshold (Figure 1A), decrease in rheobase (Figure 1B), and increase in the response to suprathreshold current injection (Figure 1C and D). Because persistent changes in excitability appeared to be restricted to IB4+ neurons, we focused on IB4+ neurons for the remainder of the study. As there were no significant differences between IB4+ neurons from naïve and inflamed rats with respect to resting membrane potential or input resistance (Table 1) and the increase in membrane capacitance, would work to attenuate excitability, we next assessed active electrophysiological properties to begin to identify mechanisms that may have contributed to the inflammation-induced increase in the excitability of IB4+ neurons. Of the active properties assessed, the action potential duration was the only property that was significant different between groups, and it was significantly longer in neurons from inflamed rats (Figure 1E, p < 0.01). There was also a trend toward an increase in the decay rate of the afterhyperpolarization (AHP) (Figure 1F, p = 0.06) in cutaneous neurons from inflamed rats. Given the role of K^+^ currents in both the action potential duration and the afterhyperpolarization (AHP), these changes in active electrophysiological properties are consistent with a decrease in a K^+^ current.

### Inflammation has no detectable influence on Kv currents in cutaneous DRG neurons

**Figure 2:**
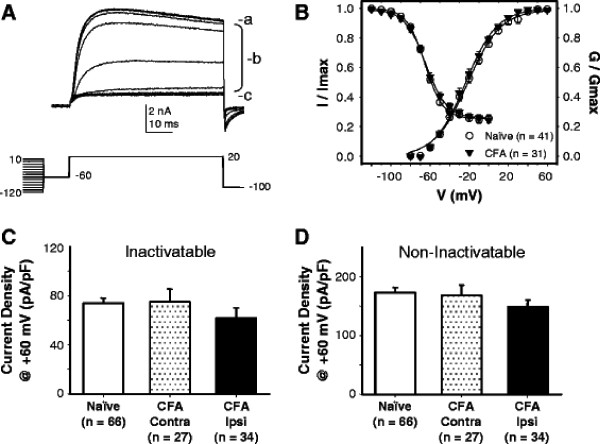
**There is no detectable influence of inflammation on voltage-gated K**^**+**^**(Kv) currents in cutaneous DRG neurons.** A. Typical Kv currents evoked in a cutaneous DRG neuron from a naïve rat with the inactivation protocol shown below the current traces. A significant fraction of total current (a) was subject to steady-state inactivation (b, inactivatable), leaving a sustained, non-inactivatable current (c). The potential at which steady-state inactivation was complete was used as the prepulse potential with which inactivatable current was separated from non-inactivatable current. B. The voltage-dependence of total Kv current activation, determined from conductance-voltage (GV) plots, was comparable in neurons from naïve and inflamed (CFA) rats. Similarly, the steady-state inactivation curves from these two groups of neurons were also comparable. Complete data sets were only collected on a subpopulation of the total number of neurons studied. C. The current density (peak current at +60 mV) of the inactivatable fraction of the total current was comparable in neurons from naïve and inflamed (CFA) rats, whether data were collected from neurons contralateral (CFA Contra) or ipsilateral (CFA Ipsi) to the site of inflammation. D. Similar results were obtained with the analysis of current density of the non-inactivatable fraction of the total Kv current in cutaneous DRG neurons

To determine whether a decrease in voltage-gated K^+^ (Kv) current contributed to the increase in excitability of cutaneous IB4+ neurons, we assessed the biophysical properties of Kv currents in cutaneous neurons from inflamed and naïve rats. Kv currents were recorded in Ca^2+^ free bath solution containing 2.5 mM Co^2+^ to eliminate contribution from BK_Ca_ channels. Based on previous data indicating that there are Kv currents both subject, and resistant, to steady-state inactivation [16], we first assessed steady-state inactivation of Kv currents in each neuron studied (Figure 2A, B). With this protocol, it was possible to determine the total current available for activation, as well as the fractions of total current subject to (inactivatable), or resistant to (non-inactivatable), steady-state inactivation. There was no detectable influence of inflammation on either the voltage-dependence of steady of inactivation or the fraction of current resistant to steady-state inactivation (Figure 2B, Table 2). Nor was there a detectable influence of inflammation on the voltage-dependence of current activation (Figure 2B). Furthermore, analysis of the peak current density of the total (not shown), inactivatable (Figure 2C) and non-inactivatable (Figure 2D) current in cutaneous neurons from naïve rats as well as both ipsilateral and contralateral to the site of inflammation revealed no significant differences between groups (p > 0.05). Finally, there were no significant differences between cutaneous neurons from naïve and inflamed rats with respect to the rate of Kv current activation, inactivation, or deactivation (data not shown). Despite evidence for inflammation-induced changes in Kv current in other populations of neurons [9], results of this series of experiments suggest that this is not the case for cutaneous neurons.

**Table 2:** Kv Current Properties

Group	N	Cap (pF)	Imax (pA/pF)	Slope (pA-mV)	V0.5 (mV)	a
Naïve	66 (7)	40 ± 1.8	166 ± 10.7	8.3 ± 0.5	-65 ± 1.0	28 ± 1.7
CFA – Contra.	27 (7)	41 ± 3.4	161 ± 14.6	11.5 ± 1.4	-65 ± 1.9	27 ± 2.4
CFA – Ipsi.	34 (7)	44 ± 2.4	160 ± 17.3	9.5 ± 1.0	-67 ± 1.7	24 ± 3.6

N is the number of neurons studied with the number of animals indicated in parenthesis. Cap is capacitance. Imax is maximal current estimated from steady-state inactivation data fitted with a Boltzmann function. Slope, V0.5 and a are parameters determined from the same Boltzmann function where Slope is the slope of the I-V curve, V0.5 is the potential at which half of the inactivatable current is inactivated, and “a” is the percentage of total current resistant to steady state inactivation.

### Inflammation results in a decrease in BK_Ca_ currents in cutaneous DRG neurons

**Figure 3:**
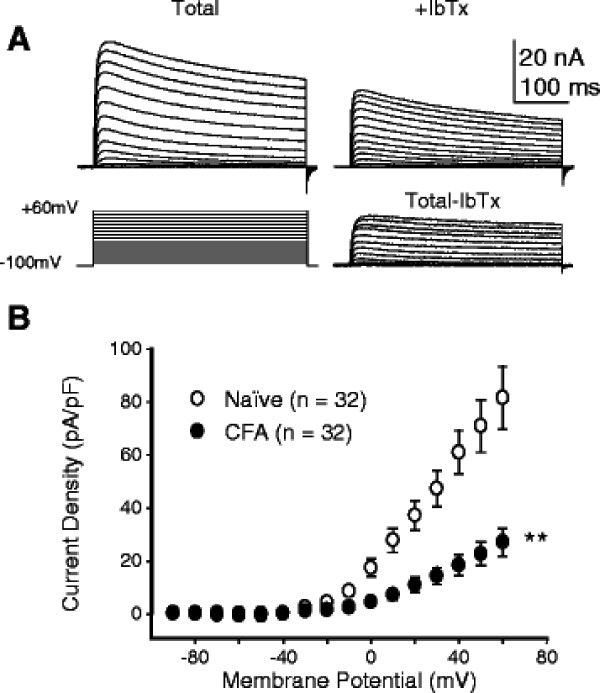
**Inflammation is associated with a significant decrease in BK**_**Ca**_**currents in cutaneous DRG neurons.** A. Outward currents were evoked before (Total) and after the application of iberiotoxin (IbTx, 100 nM), with the voltage protocol shown below the Total current traces. The toxin sensitive current (the bottom set of current traces) was obtained by digitally subtracting current evoked in the presence of IbTx from that evoked prior to its application. B. Pooled current voltage (I-V) data from cutaneous neurons from naïve and inflamed (CFA) rats highlight the significant decrease in BK_Ca_ current density in neurons from inflamed rats. Note, pooled data are only from neurons in which BK_Ca_ current was detectable (> 200 pA at +60 mV). ** is p <0.01

Based on evidence that large conductance Ca^2+^-modulated K^+^ (BK_Ca_) currents contribute to action potential duration as well as the decay of the AHP [7,8,17], as well as evidence that inflammation results in a decrease in high threshold voltage-gated Ca^2+^ channels in cutaneous DRG neurons, we next assessed the impact of inflammation on BK_Ca_ currents in cutaneous neurons. Iberiotoxin (IbTx, 100 nM) or paxilline (10 μM) were used to isolate BK_Ca_ currents from the total current evoked in IB4+ cutaneous neurons from naïve and inflamed rats (Figure 3). The IbTx sensitive current was significantly smaller in cutaneous neurons from inflamed rats than from naïve rats (Figure 3), at voltage steps to potentials ≥ −10 mV. Comparable results were obtained with paxilline, where peak paxilline sensitive current in IB4+ neurons from naïve rats (153.3 ± 28.07 pA/pF, n = 17) was significantly (p < 0.01) larger than that in cutaneous neurons from inflamed rats (49.5 ± 18.04 pA/pF, n = 10).

**Figure 4:**
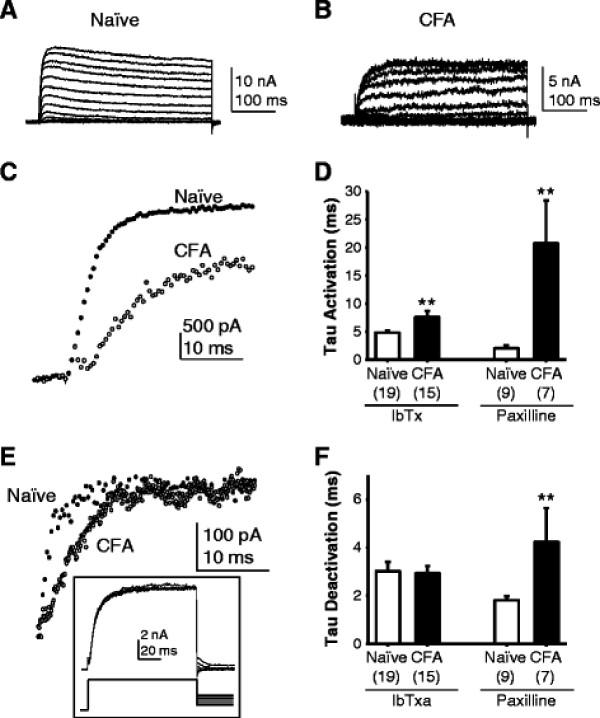
**The biophysical properties of BK**_**Ca**_**currents are altered in neurons from inflamed rats.** BK_Ca_ currents from naïve (A) and inflamed (B, CFA) rats were isolated as described in Figure 3. C. To illustrate differences in the rate of current activation, current evoked at +30 mV in the traces shown in A and B have been scaled relative to peak current and overlaid. The time constant for current activation was used to quantify the rise time. D. Pooled data indicate that this difference in activation rate is significant for currents isolated with IbTx and paxilline. E. Deactivation rate was determined from tail currents evoked at −90 mV following BK_Ca_ current activation at +30 mV. While there was no apparent difference in the deactivation rate of currents isolated with IbTx between naïve and inflamed neurons (not shown), currents isolated with paxilline appeared to deactivate more slowly in neurons from inflamed rats. Inset: example of IbTx sensitive tail currents used to assess deactivation rate. F. Pooled deactivation rate data (at -90 mV) for BK_Ca_ currents isolated with IbTx and paxilline from naive and inflamed rats. ** is p < 0.01

As previously described [8], BK_Ca_ currents in cutaneous neurons from naïve rats were in general, rapidly activating, with a variable degree of inactivation during sustained depolarization (Figure 4A). BK_Ca_ currents evoked in neurons from inflamed rats appeared to activate more slowly and demonstrated little, if any detectable inactivation during a 500 ms depolarizing voltage step (Figure 4B). Consistent with this impression, the average time constant for IbTx sensitive current activation was significantly larger in neurons from inflamed rats than in neurons from naïve rats (Figure 4C, D). Comparable results were obtained with paxilline. Deactivation of toxin sensitive currents was assessed with a tail current protocol evoked before and after toxin application (Figure 4E inset). While there was no influence of inflammation on the deactivation of IbTx sensitive currents, paxilline sensitive currents in cutaneous neurons from inflamed rats deactivated significantly more slowly than those from naïve rats (Figure 4E, F).

### Inflammation was associated with no detectable change in BK_Ca_ channel subunits expression

While the decrease in high threshold voltage-gated Ca^2+^ current previously described [6] could be sufficient to account for the inflammation-induced decrease in BK_Ca_ current density, two experiments were performed to begin to assess the possibility that a decrease in channel protein also contributed to the decrease in current. The first was a real time PCR analysis of BK_Ca_ subunit mRNA levels in L4/5 ganglia. GAPDH was used as an internal comparator as Ct values for amplification of GAPDH were comparable for ganglia from naïve (17.0 ± 0.2, n = 4) and inflamed (17.5 ± 0.3, n = 4) rats. No significant changes in mRNA levels were detected for either total α subunit or a splice variant of the α subunit containing the STREX insert, nor were there significant changes detected in mRNA levels for β2, 3 or 4: values for 2^ΔΔCt^ were all close to 1.

### Inflammation was associated with no detectable change in total protein levels of BK_Ca_ channel subunits

**Figure 5:**
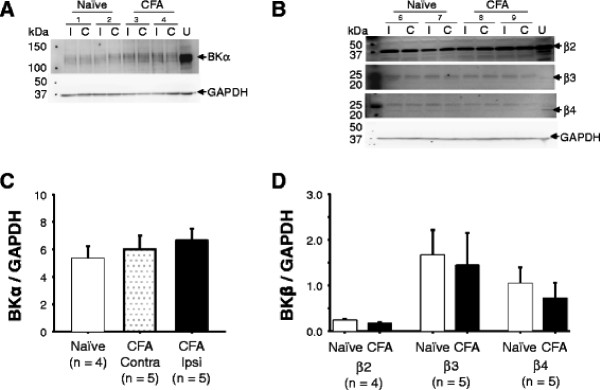
**Inflammation was associated with no detectable change in total BK**_**Ca**_**channel subunit protein levels.** Total protein was extracted from L4 and L5 ganglia from naïve and inflamed rats ipsilateral (I) or contralateral (C) to the site of CFA injection. A. Example of a western blot of the BK_Ca_ α subunit. A sample of total protein from the uterus of a naïve rat was used as a loading control to enable comparisons between blots. Ispilateral and contralateral in the naïve rats were simply left and right, respectively, as all CFA injections in the inflamed group were made in the left hindpaw. GAPDH was used as a loading control. B. Example of western blots for BK_Ca_ β subunits 2, 3, and 4. The uterus protein sample was again used to enable comparisons between blots. Pooled data for relative levels of α (C), and β (D) subunit revealed no significant differences between groups

Given evidence for inflammation-induced changes in protein in the absence of detectable changes in mRNA [18], the second experiment was to assess changes in BK_Ca_ subunit protein levels. Total protein from L4 and L5 ganglia from naïve (n = 4) and inflamed (n = 5) rats was recovered, processed for Western blot analysis, and probed with antibodies specific to the α and β2-4 BK_Ca_ channel subunits. The multiple bands present in the blots of the α subunit are consistent with the results of our previous PCR analysis of the BK_Ca_ channel in DRG which indicated that a number of splice variants of the α subunit are expressed [8]. Nevertheless, there was no detectable influence of inflammation on relative BK_Ca_ subunit protein levels (Figure 5).

### Inflammation was associated with a change in the pattern of expression of BK_Ca_ α-subunit splice variants and β-subunits

**Figure 6:**
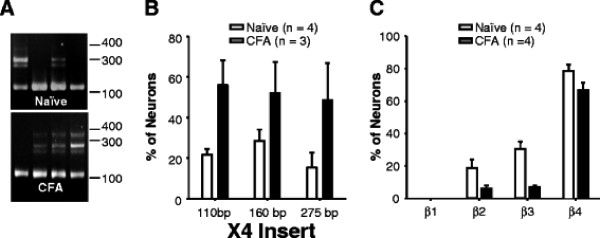
**Inflammation-induced changes in the pattern of BK**_**Ca**_**α-subunit splice variant and β subunit expression.** A. Example of splice variants at the X4 site of the BK_Ca_ α subunit detected in mRNA harvested from single cutaneous DRG neurons. Each lane is the PCR product from a single neuron. The “zero insert” bottom band is dominant in all neurons. However, the larger inserts were more common in neurons from inflamed (CFA) than naïve rats. B. Pooled data for the expression of splice variants at the X4 site from 4 naïve and 4 inflamed rats were analyzed with a two-way ANOVA, which revealed a significant influence of inflammation (p < 0.05), but no interaction between inflammation and splice variant. C. Pooled data for β subunit expression from 4 naïve and 4 inflamed rats was also analyzed with a two-way ANOVA, which also revealed a significant influence of inflammation (p < 0.05), but no interaction between inflammation and subunit expression

In our previous analysis of BK_Ca_ currents in cutaneous DRG neurons, we noted the presence of considerable heterogeneity in the biophysical properties of currents between neurons, with variability in the rate of current activation as well as in the rate and extent of current inactivation [8]. In neurons from inflamed rats, the currents were considerably more homogeneous with properties similar to those illustrated in Figure 3. Given the influence of both splice variants in the α-subunit [19], and β-subunits [20] on the biophysical properties of BK_Ca_ currents, this change raised the possibility that the changes in biophysical properties were due to changes in the subunit expression pattern. Subunit expression in cutaneous neurons was assessed with single cell RT-PCR (Figure 6). Results of this analysis indicated that there was a significant increase in the proportion of neurons in which all 4 splice variants (Figure 6, zero insert, as well as all 3 higher molecular weight species) were detected at the X4 splice site. This site corresponds to the stress-axis regulated exon (STREX) site previously described by others [20]. There was also a significant decrease in the proportion of neurons in which β2 and β3 subunits were detected.

## Discussion

### Summary of major findings

Consistent with previous studies, persistent inflammation results in an increase in the excitability of sensory neurons innervating the site of inflammation that is detectable in acutely isolated sensory neurons. This increase in excitability was restricted to the IB4+ subpopulation of neurons and was associated with a significant increase in action potential duration with no additional changes in any other passive or active electrophysiological property. These changes in excitability and action potential waveform were not associated with any detectable change in Kv current. However, they were associated with a significant decrease in IbTx- and paxilline-sensitive current. This decrease in current was not associated with a change in total BK_Ca_ subunit mRNA or protein as assessed at the whole ganglion level. Furthermore, the decrease in IbTx-and paxilline-sensitive current was associated with changes in the biophysical properties of the current as well as changes in the pattern of splice variant expression of the BK_Ca_ channel α subunit and a decrease in the proportion of neurons in which the β2 and 3 subunits were detected. These results are consistent with the suggestion that a decrease in BK_Ca_ current contributes to the inflammation-induced sensitization of cutaneous afferents.

The pattern of changes in excitability observed in cutaneous neurons were comparable to patterns observed in afferents innervating other target tissue including muscle [9], joint [21] and bladder [11]. However, there appear to be subtle, yet potentially important differences in the associated changes in passive and active electrophysiological properties. For example, the only significant change in the passive or active electrophysiological properties associated with the inflammation-induced sensitization of temporomandibular joint afferents is a decrease in the duration of the AHP [21], while in bladder afferents, there is an increase in membrane capacitance with no change in action potential duration [11]. Because passive and active properties reflect the actions of ion channels, these differences suggest differences in underlying ionic mechanisms. These differences also highlight the fact that comparable increases in excitability can be achieved through a number of different mechanisms.

### Persistent inflammation has no detectable influence on Kv currents in cutaneous neurons

Changes in passive and active electrophysiological properties assessed in current clamp can be used to predict the ion channels that contribute to the inflammation-induced increase in excitability. For example, the absence of a detectable change in V_rest_ or R_in_ argues against a channel active at the resting membrane potential such as the Cl^-^ conductance activated by inflammatory mediators we recently described in dural afferents [22]. Similarly, the increase in action potential duration and trend toward a decrease in duration of the AHP in association with a decrease in rheobase and action potential threshold are consistent with a decrease in a K^+^ current activated with membrane depolarization that contributes to membrane repolarization on the falling phase of the action potential as well as the duration of the AHP. However, in contrast to masseter muscle [9] and bladder [9] afferents, where an inflammation-induced decrease in Kv current subject to steady-state inactivation was readily detectable, no changes in Kv currents were detected in cutaneous neurons. These results suggest that a decrease in a Ca^2+^ dependent K^+^ current is likely to contribute to the inflammation-induced increase in the excitability of cutaneous neurons.

### Persistent inflammation is associated with a reduction in BK_Ca_ currents in cutaneous neurons

Consistent with the prediction based on the negative results obtained with Kv currents, we observed significant decrease in IbTx and paxilline sensitive current. The observation that the paxilline sensitive currents were larger than the IbTx sensitive currents is consistent with our single cell PCR data suggesting that the β4 subunit is present in the majority of IB4+ cutaneous neurons and previous data suggesting that the β4 subunit confers resistance to IbTx [23] but not paxilline [24]. It is important to point out that a decrease in BK_Ca_ current was not necessarily a foregone conclusion. That is, in addition to the inflammation-induced decrease in voltage-gated Ca^2+^ currents recently described in cutaneous neurons [6], there is also an inflammation-induced increase in the magnitude and decrease in the decay of the depolarization-evoked increase in the concentration of intracellular Ca^2+^ ([Ca^2+^_i_) in cutaneous neurons [5]. BK_Ca_ channels coupled to the latter process would have resulted in an increase in channel activity and a decrease in excitability [17]. However, the observed decrease in BK_Ca_ current suggests that the activation of these channels in cutaneous afferents is tightly coupled to an increase in intracellular Ca^2+^ mediated via Ca^2+^ influx through voltage-gated Ca^2+^ channels. Consistent with this suggestion is the observation that a decrease in high threshold Ca^2+^ current alone is sufficient to increase excitability, at least in a subpopulation of cutaneous afferents [8]. The observation that BK_Ca_ channels are largely restricted to IB4+ small diameter cutaneous neurons [8] is also consistent with this suggestion given the observation that the inflammation-induced increase in excitability was restricted to this subpopulation of neurons despite our previous observation that the inflammation-induced decrease in voltage-gated Ca^2+^ currents is detectable in both IB4+ and IB4- neurons [6].

The absence of a detectable inflammation-induced change in BK_Ca_ channel subunit mRNA or protein at the whole ganglia level is also consistent with the suggestion that the decrease in BK_Ca_ current was largely secondary to the decrease in voltage-gated Ca^2+^ current rather than a decrease in BK_Ca_ channels. This suggestion is made with caution, however, given that afferents innervating the site of inflammation constitute far less than half of the neurons in L4 and L5 ganglia and as a result, a decrease in mRNA or protein in a subpopulation of these neurons may not have been detectable.

### Changes in BK_Ca_ subunit expression are associated with inflammation-induced changes in the biophysical properties of BK_Ca_ currents in cutaneous neurons

Changes in the biophysical properties of the BK_Ca_ currents in neurons from inflamed rats suggest that a decrease in high threshold voltage-gated Ca^2+^ current alone is insufficient to account for all of the changes in BK_Ca_ currents. At least some of the changes in biophysical properties appear to reflect changes in the pattern of BK_Ca_ channel subunit expression. That is, β3 subunits confer rapid channel activation, and β2 confers both rapid channel activation and channel inactivation [20]. Thus, a decrease in the number of cutaneous neurons in which these subunits were detected is consistent with the more slowly activating persistent currents observed in neurons from inflamed animals. However, at least two of the inserts at the X4 site of the BK_Ca_ α subunit result in a dramatic leftward shift in the voltage-dependence of channel activation [19], an increase in the rate of channel activation as well as a decrease in the rate of channel deactivation. The decrease in rate of paxilline-sensitive, but not IbTx-sensitive current deactivation suggests there may be preferential assembly of channels with an X4 insert in the α-subunit and a β4 subunit (resulting in a channel with a slower deactivation rate that is resistant to IbTx), but additional biochemical data would be needed to confirm this prediction. Several possibilities could account for the failure to detect a larger influence of the splice variants on the whole cell current. These include: 1) that the larger α subunit transcripts are not translated as efficiently, 2) that the larger α subunits are not assembled in inflamed neurons, or 3) that the trafficking of these larger channels is also altered such that they are targeted to other parts of the afferent than the cell soma. These latter two possibilities are predicated on the assumption that at least some of the variability in biophysical properties of BK_Ca_ currents observed in neurons from naïve animals [8] is due to the presence of splice variants of the α subunit.

## Conclusions

A decrease in K^+^ current is a general mechanism found throughout the nervous system to increase in neuronal excitability. The ubiquity of this mechanism begs the question as to why there should be diversity in the specific channels that are reduced in primary afferent neurons as a function of the target of innervation and or the type of injury [1]. It is certainly possible that because the mediators such as prostaglandin E2, TNFα and nerve growth factor, that drive the changes in K^+^ channels have specific intracellular targets, and that different K^+^ currents are suppressed in different populations of afferents because the pattern of mediators varies with target of innervation and type of injury. This possibility does not account for the fact that the net result in each case is an increase in excitability. Given the impact of subtle differences in K^+^ channels properties on spiking behavior, the primary impact of this diversity is likely to be net differences in neuronal activity with some channels favoring one type of output such as sustained or bursting activity, whereas other channels favoring irregular activity. Of course the context in which these changes take place will also be critical for the net change in output [25], where the biophysical properties, density and distribution of other ion channels in the neuron will also impact the net change in afferent output. Minimally, this level of diversity serves as a reminder as to why it has been so difficult to identify more effective therapeutics for the treatment of pain devoid of deleterious consequences. This is also additional support for the suggestion that the most effective therapeutic interventions may ultimately need to be tailored to the specific site and type of injury.

## Methods

Adult male Sprague–Dawley rats (Harlan Sprague Dawley, Indianapolis IN) were used for all experiments. Rats were housed in an AAALAC approved animal facility with a 12:12 light/dark cycle (lights off at 7 PM) with food and water available *ad libitum*. All procedures were approved by the University of Pittsburgh Institutional Animal Care and Use Committee and performed in according with the recommendations of the National Institutes of Health and the Committee for Research and Ethical Issues of the International Association for the Study of Pain. All efforts were employed to minimize the number of animals used in this study.

### Retrograde labeling and inflammation

Cutaneous afferents were retrogradely labeled with DiI as previously described [5,8]. Briefly, rats were anesthetized with isofluorane and 10 μl of 1,1’-dioctadecyl-3,3,3’,3’-tetramethylindocarbocyanine perchlorate (DiI, Invitrogen, Carlsbad CA, 17 mg/ml in saline diluted from a stock of 170 mg/ml in DMSO) was injected at 3–5 sites (with 1.5 - 2 μl per site) with a 30 g needle directed into the epidermis. Rats were studied 14 to 17 days post DiI injection. Inflammation was induced in a subgroup of rats with a 100 μl subcutaneous injection of complete Freund’s adjuvant (CFA, mixed 1:1 in saline) into the same site previously labeled with DiI. This injection was also made under isofluorane-induced anesthesia. This group of rats was studied 3 days after CFA injection.

### Preparation of isolated cutaneous neurons

Acutely dissociated cutaneous neurons were obtained as previously described [5,8]. Briefly, rats were deeply anesthetized with 1 ml/kg rat cocktail (55 mg/ml ketamine, 5.5 mg/ml xylazine and 1.1 mg/ml acepromazine) and L4 and L5 dorsal root ganglion (DRG) were harvested, enzymatically treated, mechanically dissociated and plated onto laminin-ornithine coated cover slips. After 2 hrs of incubation at 37°C/3% CO_2_, cover-slips were flooded with HEPES buffered L-15 media and stored at room temperature during the period of recording (< 8 hrs after removal from the animal).

### Patch clamp recording

Isolated cutaneous neurons were studied with conventional whole-cell and perforated patch configurations with an Axopatch 200B (Medical Devices Sunnyvale CA) controlled with pClamp (v 8.2, Molecular Devices) or a HEKA EPC9 amplifier (HEKA Electronik, Lambrecht/Pfalz Germany) controlled with Pulse software (V8.8, HEKA). The conventional whole cell configuration was used for current clamp recording and for voltage-clamp analysis of Kv currents while the perforated patch configuration was used for the analysis of BK_Ca_ currents. For current clamp recording, the bath solution contained (in mM): NaCl, 130; KCl, 5; CaCl_2_, 2.5; MgCl_2_, 0.6; HEPES, 5; and glucose, 10; pH adjusted to 7.4 with Tris-Base, and osmolality adjusted to 320 mOsm with sucrose. For voltage-clamp recording of Ca^2+^ dependent K^+^ currents, NaCl was replaced with choline-Cl to eliminate voltage-gated Na^+^ currents. This same solution was used for voltage clamp recording of voltage-gated K^+^ currents, except that CaCl_2_ was replaced with CoCl_2_ to eliminate voltage-gated Ca^2+^ currents. The electrode solution used for current clamp and to record voltage-gated K^+^ currents contained (in mM): KCl, 30; K-methanesulfonate (MES), 110; MgCl_2_,1; CaCl_2_, 0.1; EGTA, 1; HEPES, 10; ATP-Mg, 2; GTP, 1; pH adjusted to 7.2 with Tris-Base, and osmolality adjusted to 310 mOsm with sucrose. The electrode solution used for perforated patch clamp recordings contained (in mM): KCl, 30; K-MES, 110; MgCl_2_, 1; HEPES, 10; EGTA, 0.1; pH adjusted to 7.2 with Trisbase, and osmolality adjusted to 310 mOsm with sucrose. Amphotericin B, used to obtain whole cell access for perforated patch recording, stock solution was prepared in DMSO (90 mg/ml) then diluted to a final concentration (600 μg/ml) in electrode solution immediately prior to use. All salts used for electrophysiology were obtained from Sigma-Aldrich (St Louis MO).

### Semi-quantitative RT-PCR (sqRT-PCR)

Dorsal root ganglia (DRG) from anesthetized male rats were harvested in a manner identical to that used for neuron isolation and plating. mRNA was extracted and cDNA synthesis performed as previously described [6], except that that random hexomers were used to prime the reverse transcription reaction. SYBR Green was used to monitor amplification of template with primers on a real-time thermal cycler (Life Technologies, Grand Island NY) controlled by a PC running Prism 7000 SDS software. A melting curve was generated at the end of each experiment to assess for the presence of contamination. Amplification efficiency was determined for each target gene. The ΔΔCT method was used to assess differences in relative expression levels. Primers for amplification of the core BK_Ca_ α subunit were: F - TGTCATGATGACGTCACAGATCC, R -TTTTTTTGGTGACAGTGTTGGC; those for amplification of the BK_Ca_ α subunit with the STREX insert were: F - AGCCGAGCATGTTGTTTTGAT, R- ACGCACACGGCCTGACA; while those for GAPDH were: F - GGCCTACATGGCCTCCAA, R -TGGAATTGTGAGGGAGATGCT. Commercially available primer sets were used for the amplification of β2, 3 and 4 (Qiagen).

### Single cell PCR

Single cell PCR was performed as previously described [26]. Because perforated patch recording is relatively slow and many neurons are needed from a single animal to obtain a reasonable estimate of the proportion of neurons from a single animal, a different set of neurons was used for single cell PCR analysis. Following identification of cutaneous neurons under epifluorescence illuminations, neurons were collected with large bore (~30 μm) glass pipettes and expelled into microcentrifuge tubes containing reverse transcriptase (RT) mix. RT-PCR was performed as described previously [26] utilizing an anchored primer (5’-ttttttttttttttttttvn-3’; v = a, c, or g; n = a, c, g, or t, from Life Technologies) for the RT reaction and a nested PCR amplification strategy for the PCR reaction. rslo primer sequences were identical to those described previously [8]. For each cell preparation, at least two tubes were run in which no cell was collected (although the electrode was manipulated in a manner identical to that used for cell collection) and at least two additional tubes were run in which no reverse transcriptase was added to the RT mix prior to the RT reaction. Cyclophillin (0.5 μl of cDNA) was used to monitor the success of the cell collection/RT reaction: only neurons in which cyclophillin was detected were used for further analysis. 5 μl of PCR products were loaded onto 2% agorase/TAE gel.

### Western Blot

DRG (L4/L5) were rapidly removed from deeply anesthetized rats and homogenized in solubilization buffer (50 mM Tris.HCl, pH8.0; 150 mM NaCl, 1 mM EDTA, 1% NP40, 0.5% deoxycholic acid, 0.1% SDS, 1 mM Na_3_VO_4_, 1 U/ml aprotinin, 20 μg/ml leupetin, 20 μg/ml pepstatin A). The homogenate was centrifuged at 20,000 X g for 10 min at 4°C. The supernatant was removed. Protein (50–120 μg) was separated on a 7.5-10% SDS-PAGE gel and blotted to nitrocellulose membrane (Amersham) with a Trans-Blot Transfer Cell system (Bio-Rad). Blots were blocked with 5% milk in TBS buffer (20 mM Tris, 150 mM NaCl pH 7.4) at room temp for 1 hour. After decanting the blocking buffer, the blots were incubated with primary antibodies. These included: the α subunit of the BK_Ca_ channel (AKA slo1, NeuroMab clone L6/60, NeuroMab, Davis CA: 1:200), BK_Ca_ β2 (NeuroMab clone N53/32: 1:200), BK_Ca_ β3 (NeuroMab clone N40B/18: 1:200), BK_Ca_ β4 (NeuroMab clone L18A/3: 1:200), and GAPDH (sc-25778, Santa Cruz Biotechnology: 1:1000). The specificity of all BK_Ca_ subunit antibodies has been confirmed in heterologous expression systems where there was no evidence of cross reactivity with other BK_Ca_ subunits or KV2.1. Membranes were incubated with BK_Ca_ subunit antibodies overnight at 4°C, and GAPDH for 1 hour at room temperature. Membranes were washed with TBS buffer and incubated for 1 hour with anti-goat IgG horseradish peroxidase (1:3000, Santa Cruz) in 5% milk/TBS. Membranes were then washed with TBS buffer. The immunoreactivity was detected using Enhanced Chemiluminescence (ECL, Amersham). Chemiluminescence was captured with a CCD camera (Las-3000, Fujifilm) and analyzed with Fuji software Multi Gauge. The relative protein levels were obtained by comparing target protein to loading control (GAPDH) in the same membrane.

### Statistical analysis

Data are expressed as mean ± S.E.M unless otherwise stated. Student’s *t* test, one- and two-way ANOVA with the Holm-Sidak post hoc test were used for comparisons of parametric data between groups. For single cell PCR analysis, between 30 and 40 cutaneous neurons were collected from each animal, although only 29 neurons were collected for one of the 4 naïve animals used to assess changes in α-subunit splice variants. The proportion of the total number of neurons from each animal in which a splice variant or β-subunit was detected was used as the “statistic” for that animal, where the mean proportion of expression in naïve animals was compared to that in inflamed animals. Statistical significance was assessed at p < 0.05.

## Abbreviations

AHP: Afterhyperpolarization; AP: Action potential; BKCa: Iberiotoxin or paxilline sensitive large conductance Ca2+ modulated K+ channel; CFA: Complete Freund’s adjuvant; DRG: Dorsal root ganglion; IbTx: Iberiotoxin; Kv: Voltage-gated K + current; PCR: Polymerase chain reaction; STREX: Stress axis regulated exon.

## Competing interests

None of the authors have either financial or non-financial competing interests in relation to any of the material described in this manuscript.

## Authors’ contributions

XLZ carried out the electrophysiological studies and drafted the manuscript. LM validated the strategy to detect multiple splice variants from a single cell and carried out the molecular biological analysis. KYL contributed to the electrophysiological analysis. MC carried out western blot analysis. MSG conceived of the study, and participated in its design and coordination and analysis and helped to draft the manuscript. All authors read and approved the final manuscript.

## Authors’ information

Dr. Zhang is presently at the University of Pittsburgh in the Department of Pharmacology, 200 Lothrop Street Room E1303 BST, Pittsburgh PA 15213. Ms. Mok is presently at the Division of Biology, California Institute of Technology, 1200 East California Boulevard, Pasadena, CA 91125. Mr Charbonnet is presently at the Hayward Genetics Center, Tulane University School of Medicine, 1430 Tulane Avenue, Box SL-31 New Orleans, LA, 70112.
